# Improving the Gelation Properties of Pea Protein Isolates Using Psyllium Husk Powder: Insight into the Underlying Mechanism

**DOI:** 10.3390/foods13213413

**Published:** 2024-10-26

**Authors:** Qiongling Chen, Jiewen Guan, Zhengli Wang, Yu Wang, Xiaowen Wang, Zhenjia Chen

**Affiliations:** 1 College of Food Science and Engineering, Shanxi Agricultural University, Jinzhong 030801, China; cql_ttxs@163.com (Q.C.); guan15536795286@163.com (J.G.); 18534272895@163.com (Z.W.); sxtgwy@126.com (Y.W.); wwxw11@163.com (X.W.); 2 Shanxi Province Innovation Center for Storage and Processing Technology of Fruit and Vegetable, Jinzhong 030801, China

**Keywords:** pea protein, psyllium husk powder, protein gel, conformational change, gelation mechanism

## Abstract

The industrial application of pea protein is limited due to its poor gelation properties. This study aimed to evaluate the effects of psyllium husk powder (PHP) on improving the rheological, textural, and structural properties of heat-induced pea protein isolate (PPI) gel. Scanning electron microscopy (SEM), intermolecular forces analysis, the quantification of the surface hydrophobicity and free amino groups, and Fourier transform infrared spectroscopy (FTIR) were conducted to reveal the inner structures of PPI-PHP composite gels, conformational changes, and molecular interactions during gelation, thereby clarifying the underlying mechanism. The results showed that moderate levels of PHP (0.5–2.0%) improved the textural properties, water holding capacity (WHC), whiteness, and viscoelasticity of PPI gel in a dose-dependent manner, with the WHC (92.60 ± 1.01%) and hardness (1.19 ± 0.02 N) peaking at 2.0%. PHP significantly increased surface hydrophobicity and enhanced hydrophobic interactions, hydrogen bonding, and electrostatic interactions in PPI-PHP composite gels. Moreover, the electrostatic repulsion between anionic PHP and negatively charged PPI in a neutral environment prevented the rapid and random aggregation of proteins, thereby promoting the formation of a well-organized gel network with more β-sheet structures. However, the self-aggregation of excessive PHP (3.0%) weakened molecular interactions and disrupted the continuity of protein networks, slightly reducing the gel strength. Overall, PHP emerged as an effective natural gel enhancer for the production of pea protein gel products. This study provides technical support for the development of innovative plant protein-based foods with strong gel properties and enriched dietary fiber content.

## 1. Introduction

The burgeoning demand for plant protein-based foods, driven by increasing consumer awareness of health benefits, environmental sustainability, and animal welfare concerns, is reshaping the global food landscape, as evidenced by exponential market growth [1]. Among the various plant proteins available, pea protein has emerged as a competitive resource with numerous advantages, including widespread availability, a balanced essential amino acid composition, high lysine content, low odor, low allergenicity, and non-genetic modification [2]. Pea protein has been widely used in a variety of food formulations, such as bread, pasta, meat products, and beverages, to increase protein content in diets [3,4]. Additionally, it serves as a binder, emulsifier, stabilizer, or extender in foods [4]. However, pea protein exhibits poor gelling properties due to its limited solubility, the low proportion of cysteine involved in disulfide bond generation, and its susceptibility to thermal denaturation [5]. Additionally, the formation of pea protein gel requires high temperature and high concentration conditions [6]. These unfavorable gelation characteristics of pea protein greatly restrict its application in the food industry.

**Figure 1:**
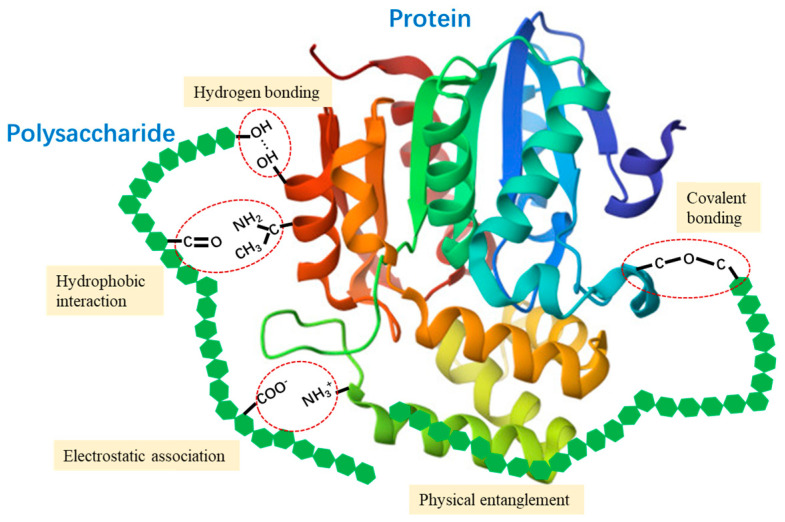
Schematic diagram of molecular interactions between protein and polysaccharide.

To address these limitations of pea protein in gelation properties, researchers have turned to polysaccharides as natural texture enhancers and effective modifiers of pea protein gel. During heat-induced gelation, polysaccharides could interact with proteins through electrostatic associative interactions, hydrogen bonding, hydrophobic interactions, and covalent bonding (Figure 1). These interactions modulate the rheological behaviors and alter the conformational structures of proteins. Consequently, the physical entanglement and intermolecular interactions between proteins and polysaccharides facilitates the formation of a composite protein–polysaccharide network with improved gel texture and stability [7]. Moreover, the strong water-absorbing and binding capacity of polysaccharides changes the microenvironment surrounding proteins and causes a concentrated condition for protein molecules, thus reducing the intermolecular distances and promoting protein aggregation and crosslinking [8]. Previous studies have reported that the addition of inulin [9], pectin [7], cellulose nanocrystals [2], and gellan gum [6] could effectively enhance the gel strength of pea protein isolate (PPI) gels and promote the formation of gel networks with heterogeneous and compact microstructures.

However, the enhancement effects of polysaccharides on physicochemical properties of thermally induced protein gels are directly influenced by the specific polysaccharide type, concentrations, pH, ionic strength, temperature, and other factors. Among them, the polysaccharide concentration plays a critical role in determining the assembly mode and interplay method between the protein and polysaccharide during the formation of composite gels. He, et al. [10] reported that low concentrations (≤0.5%, *w*/*v*) of pectin enhanced the textural properties of ginkgo seed protein–pectin composite gels by hydrogen bonding and appropriately modulating the charge density. Conversely, an excessive amount of pectin (1.0%, *w*/*v*) weakened the gel texture with an overfilling effect. Similarly, the study of Gao, et al. [11] indicated that an appropriate amount of cellulose nanocrystals (CNC, 0.25–0.75%), complexed with PPI subunits (legumin and vicilin) mainly via hydrogen bonds and van der Waals forces, promoted the formation of an ordered and compact three-dimensional network. However, overmuch CNC (1.00%) tended to self-agglomerate and separate from PPI due to thermodynamic incompatibility, causing the formation of a loose gel network structure with large pores. Therefore, altering the concentration of polysaccharides can cause completely different outcomes concerning phase separation behaviors, molecular interaction methods, protein aggregation patterns, and the final network structures of plant protein gels.

Psyllium husk powder (PHP), derived from the husk of psyllium seeds, has recently received considerable attention due to its clean label attribute, rich content of dietary fiber (81%), a high proportion of soluble dietary fiber (70%), multifaceted properties, and health benefits [12]. PHP is readily available and inexpensive due to its simple and solvent-free processing steps, including dehulling, meshing, and sieving. The main polysaccharide in PHP is highly branched arabinoxylan, which is predominantly composed of xylose (65%) and arabinose (20%) [13]. The polysaccharide in PHP endows it with unique functional properties, including an extremely strong water-binding capacity, remarkable viscosity enhancement, and an excellent gel-forming ability. Researchers have extensively explored the utilization of PHP as a gelling agent, texture enhancer, partial fat substitute, edible stabilizer, and dietary supplement in various foods, including yogurt, sausages, chicken patties, bread, biscuits, and cakes [14]. Previous studies have reported that the appropriate addition of PHP could greatly improve the gel strength of myofibrillar proteins from goose [12], lamb [15], pig [16], and surimi [16] through enhancing hydrogen bonding and forming interpenetrating structures in composite gels. However, limited information in the literature is available on the application of PHP in improving the gelation properties of plant proteins and their interaction mechanisms. There is still some distance to go to understand how the concentration of PHP influences the phase behavior, conformation transformation, and gelation kinetics of pea protein at the molecular level.

Therefore, the current research aims to unravel the impact of PHP with different concentrations (0%, 0.5%, 1.0%, 2.0%, and 3.0%) on characteristics of heat-induced PPI gels, including color attributes, the water holding capacity (WHC), textural properties, the visual appearance, and inner microstructures. Rheological properties were determined to clarify the effects of PHP on the hydration kinetics and viscoelastic behaviors of PPI during the gelation process. Furthermore, the underlying mechanisms were comprehensively explored in terms of conformational changes and molecular interactions. The current findings will provide technical support for expanding the application of pea proteins in various foods, such as plant-based meat analogs, fat substitutes, cheese, yogurt, sausages, desserts, confectioneries, jellies, 3D printing materials, etc. This study will also provide a theoretical reference for the utilization of PHP in developing innovative plant protein-based foods with improved texture, nutritional value, and consumer acceptance.

## 2. Materials and Methods

### 2.1. Materials

PPI, with a protein content of 81.8%, was obtained from Shuangta Food Co., Ltd. (Yantai, China). According to the manufacturer, the PPI was prepared using alkaline solubilization and isoelectric precipitation techniques followed by spray drying. PHP with a purity of 99% was acquired from Weiqing Biotechnology Co., Ltd. (Shanghai, China) following the pre-processing of milling and 100-mesh sieving. All other reagents were purchased from Sigma Chemical Co., Ltd. (St. Louis, MO, USA) and Solarbio Technology Co., Ltd. (Beijing, China), and were of analytical grade.

### 2.2. Gel Preparation

A solution of 22.5% (*w*/*v*) PPI was prepared by dissolving 4.5 g of PPI in 20 mL of deionized water. Subsequently, the PHP was suspended into the PPI dispersion with regulating concentrations at the levels of 0.5%, 1.0%, 2.0%, and 3.0% (*w*/*v*). The PPI-PHP mixed solution was stirred at room temperature for 1 h to obtain a fully dissolved and hydrated dispersion. The dispersion was heated at 95 °C for 30 min, cooled to room temperature, and stored at 4 °C for 12 h. The 22.5% PPI dispersion subjected to the same treatment was used as the control group.

### 2.3. Determination of Gel Properties

#### 2.3.1. Gel Color

The color attributes of the gel samples, including lightness (L*), redness/greenness (a*), and yellowness/blueness (b*) values, were measured using an instrumental colorimeter (YS-3060, Shenzhen ThreeNH Technology Co., Ltd., Shenzhen, China). The whiteness (W) values were calculated according to Equation (1):

(1) $$W=100-\sqrt{{\left(100-L^{*}\right)}^{2}+a^{*2}{+b}^{*2}}$$

#### 2.3.2. WHC

The composite gels were wrapped in filter paper and subjected to centrifugation at 4500 rmp for 10 min. Afterward, the centrifugal tube was inverted to drain the supernatant, and the remaining water was removed with filter paper. The weights of the samples before and after centrifugation were recorded as W_1_ and W_2_, respectively. The WHC was calculated according to Equation (2):

(2) $$WHC=\frac{W_{2}}{W_{1}}\times 100\%$$

#### 2.3.3. Textural Profile Analysis (TPA)

The texture properties of PPI-PHP composite gels were evaluated using a TMS-Pro texture analyzer (Food Technology Corporation, Sterling, VA, USA) with a 36 mm cylindrical probe under TPA mode. The composite gels with dimensions of 25 × 10 mm (diameter × height) were equilibrated at 25 °C for 1 h prior to the tests. Uniaxial double compression tests were performed at pre-test, test, and post-test speeds of 1 mm/s with a trigger force of 0.05 N, a deformation distance of 5 mm, and a gap time of 2 s. Textural parameters, including hardness, cohesiveness, springiness, and chewiness, were recorded automatically by the instrument.

#### 2.3.4. Visual Appearance and Microstructure Observation

A digital camera was used to observe and record the inner macrostructures of the composite gels. The microstructure was visualized using a field-emission scanning electron microscope (SEM, JSM-7500F, JEOL Ltd., Tokyo, Japan). The composite gels were sliced and lyophilized, then manually fractured to expose the inner cross-section. Subsequently, the samples were stuck on a metal stub and sputtered with platinum. SEM images were captured at an accelerating voltage of 5 kV.

### 2.4. Rheological Properties

The gelation process was monitored using temperature sweep tests with a rotary rheometer (Physica MCR302, Anton Paar, Graz, Austria) equipped with a 50 mm parallel plate geometry (PP50). Approximately 1.5 mL of the PPI-PHP mixed solutions (described in Section 2.2) were loaded onto the plate and sealed with a thin layer of silicone oil. The strain was maintained at 0.5% within the linear viscoelastic region, and the frequency was set constantly at 1 Hz. The measurements were performed by heating the solutions from 25 °C to 95 °C at 5 °C/min, incubating them at 95 °C for 30 min, and then cooling them down to 25 °C at 5 °C/min. The storage modulus (G′), loss modulus (G″), and loss tangent (tan δ = G″/G′) were recorded continuously.

### 2.5. Molecular Forces Analysis

The molecular interaction forces were assessed through protein solubility in five types of dissociation reagents [17]. The gel samples (2.0 g) were dissolved in 20 mL of the following solutions: (S1) 0.05 mol/L of NaCl, (S2) 0.6 mol/L of NaCl, (S3) S2 + 1.5 mol/L of urea, (S4) S2 + 8 mol/L of urea, and (S5) S4 + 0.5 mol/L of β-mercaptoethanol. After stirring at room temperature for 60 min and centrifugation at 4500 rpm for 15 min, the protein content in the supernatants was quantified by the Coomassie brilliant blue method. The relative contents of ionic bonds, hydrogen bonds, hydrophobic interactions, and disulfide bonds were calculated by the differences in protein contents in solutions (S2) and (S1), solutions (S3) and (S2), solutions (S4) and (S3), and solutions (S5) and (S4), respectively.

### 2.6. Surface Hydrophobicity

The surface hydrophobicity index (H_0_) was assessed according to the method of Sun, et al. [18] with 1-anilinonaphthalene-8-sulfonic acid (ANS) as the fluorescent probe. The samples were dissolved in 0.01 M of phosphate buffer (pH 7) and diluted serially to concentrations ranging from 0.03 mg/mL to 0.5 mg/mL. Subsequently, 20 μL of the 8 mM ANS solution was added to 4 mL of each diluted sample. The fluorescence intensity was measured using a Hitachi F2500 fluorescence photometer (Tokyo, Japan) with excitation and emission wavelengths set at 390 nm and 470 nm, respectively. The initial slope of the fluorescence intensity versus protein concentration was calculated by linear regression analysis and designated as H_0_.

### 2.7. Quantification of Free Amino Groups

The content of free amino groups was quantified using the O-phthalaldehyde (OPA) method with leucine as the standard [19]. The absorbance was recorded at 340 nm by an ultraviolet-visible spectrophotometer (UV-1201, Shimadzu, Kyoto, Japan).

### 2.8. Sodium Dodecyl Sulfate-Polyacrylamide Gel Electrophoresis (SDS-PAGE)

The subunit compositions of PPI-PHP composite gels were investigated according to the method described by Sun, Wang, Wang, Zhou, Jiang, and Zhu [18] with a Mini-PROTEAN System (Bio-Rad, Hercules, CA, USA). The gel sample solutions (4 mg/mL) were prepared by dissolving them in sample buffer (0.08 M Tris-HCl buffer, pH 6.8), 1% (*w*/*v*) SDS, 2% (*v*/*v*) β-mercaptoethanol, 5% (*v*/*v*) glycerol, and 0.025% (*w*/*v*) bromophenol blue. Then, the solutions were heated at 100 °C for 10 min, followed by centrifugation at 6000× *g* for 15 min. Electrophoresis experiments were conducted with a 10% separating gel (pH 8.8) at 80 V and a 5% stacking gel (pH 6.8) at 110 V. Subsequently, the gels were stained with a 0.1% Coomassie brilliant blue solution and destained with a solution containing 5% ethanol and 10% acetic acid.

### 2.9. Fourier-Transform Infrared Spectroscopy (FTIR)

The freeze-dried gel samples were evenly mixed and ground with KBr at a ratio of 1:100 and pressed into transparent sheets. Absorbance spectra were recorded using a Fourier-transform infrared spectrophotometer (TENSOR 27, Bruker, Karlsruhe, Germany) in the range of 400–4000 cm^−1^ with 64 scans and a resolution of 4 cm^−1^. The relative proportions of secondary structures were calculated by the Gaussian curve fitting in the amide I region (1700–1600 cm^−1^) using the PeakFit 4.12 software (Seasolve, Framingham, MA, USA).

### 2.10. Statistical Analysis

All experiments were conducted in three independent batches. Data were processed using SPSS 19.0 (SPSS Inc., Chicago, IL, USA) and expressed as the mean ± standard deviation. Significant differences (*p* < 5%) were identified using the one-way ANOVA method and Duncan’s tests. The plots of results were obtained with the Origin 19.0 software (Origin Lab Corp., Northampton, MA, USA).

## 3. Results and Discussion

### 3.1. Color Attributes

Color is one of the most intuitive characteristics of gel products, which directly influence consumers’ preference and acceptance. Table 1 shows the effects of PHP addition on the lightness (L*), redness (a*), yellowness (b*), and whiteness values of PPI-PHP composite gels. Compared with PPI gel, the L* value was significantly increased (*p* < 0.05) from 50.20 ± 0.26 to 73.34 ± 0.90 with the addition of 0.5% PHP, and it reached the highest value (76.21 ± 0.15) at 2.0% PHP. This result suggests that PPI and PHP worked synergistically to form a denser and more homogenous inner gel network, which increased the refractive index and caused stronger light scattering [20]. On the one hand, PHP competed with PPI for water, increasing the relative concentration of PPI and thus improving the probability of protein collisions and aggregation. On the other hand, PHP could act as a filler or copolymer within the protein matrix and occupy the voids in the three-dimensional network of protein gel, thereby contributing to structural reinforcement and enhancement of the firmness. The presence of PHP slightly increased the a* value from 2.17 ± 0.07 to 2.28–2.37, whereas it significantly increased the b* value from 14.04 ± 1.38 to 18.86–20.07. However, there was no significant difference in a* and b* values between the composite gels with different PHP concentrations. This result suggested that these increases in yellowness and redness may be caused by the brown pigments and the polyphenols in PHP rather than the Maillard reaction, which is generally positively correlated with the concentration of polysaccharides [21]. As the PHP content increased from 0 to 2.0%, the whiteness value significantly increased from 48.20 ± 0.19 to 68.78 ± 0.12 and then decreased to 64.81 ± 1.38 with 3.0% PHP. This trend was consistent with the changes in WHC and textural properties (in Section 3.2 and Section 3.3). These results agreed with previous findings of Yanan et al. [22] who reported that denser gel networks locked more water molecules, thus changing the light scattering effect and causing a whiter appearance. Overall, the moderate addition (0.5%–2.0%) of PHP was beneficial in the formation of PPI gels with a desirable smoother appearance and whiter color.

### 3.2. WHC

The WHC of protein gels is a critical determinant of their texture, juiciness, and overall sensory attributes. It also serves as an indirect reflection of the strength and extent of intermolecular interactions within the gel matrix, particularly hydrogen bonding and hydrophilic interactions. As indicated in Table 1, pea protein gels exhibited a poor WHC of 78.4 ± 0.7% compared to heat-induced protein gels prepared from 14% (*w*/*v*) soy protein (approximately 96%) [19], 12% (*w*/*w*) lentil protein (approximately 95%) [23], and egg white at a neutral pH (90%) [24]. The low WHC of PPI gels may be attributed to the extraction and isolation procedures of commercial PPI that induce partial protein denaturation and the formation of insoluble aggregates [25]. The existence of these insoluble aggregates can disrupt network formation by acting as inactive fillers, resulting in coarser network structures [26]. The incorporation of PHP effectively enhanced the WHC of heat-induced PPI gels. This improvement can be attributed to rich hydroxyl groups of PHP, which can bind water through the formation of hydrogen bonds. Additionally, PHP can interact with pea proteins through other non-covalent interactions or physical entanglements, thereby enhancing the structural integrity of the composite gels. Furthermore, the uniform dispersion of PHP within the protein matrix favored the generation of a highly crosslinked network structure (as shown in the SEM images), which could provide physical interception of water molecules through capillary forces. As the content of PHP increased from 0% to 2.0%, the WHC of PPI-PHP composite gels gradually increased from 78.4 ± 0.7% to 92.6 ± 1.0%. Similarly, previous studies have reported that the addition of PHP increases the WHC of goose myofibrillar protein gels and plant-based sausages in a dose-dependent manner [12,27]. As the concentration of PHP increased, more hydrophilic sites became available for water binding, thereby reducing the ease of water movement within the gel and increasing the overall WHC of the composite gels. However, the addition of PHP at a level of 3.0% caused a slight decrease in WHC. This change may be due to the fact that adding 3.0% PHP would cause thermodynamic incompatibility and phase separation between macromolecules. After heating–cooling treatment, the local gelation and self-aggregation of PHP tended to form a continuous hydrogel, which destroyed the integrity of the protein gel network and reduced its capacity to retain water. Overall, the improvement of the WHC for PPI gel by PHP was advantageous for the application of PPI in gel-type products as it facilitated modifications to the texture and an increase in product yield through improving moist binding.

### 3.3. Textural Properties

The texture characteristics of a protein gel play a pivotal role in determining its overall quality, as they directly influence sensory attributes such as the palatability, mouthfeel, and swallowing properties of food, thus significantly impacting consumer acceptance and product functionality. A two-bite test for texture profile analysis was employed to simulate the changes of PPI-PHP composite gels when they were subjected to mastication. As shown in Table 2, the incorporation of PHP remarkably increased the hardness, cohesiveness, springiness, and chewiness for the full range of additive amounts compared with the heat-induced PPI gel (*p* < 0.05), corresponding to the visual observation (in Section 3.4). The reinforcement of texture indicated that PHP participated in the gel formation process, effectively modifying and enhancing the three-dimensional network structures of PPI gel. As the PHP concentration increased from 0% to 2%, the hardness and cohesiveness of the PPI-PHP composite gel were noticeably increased from 0.65 ± 0.02 N and 0.39 ± 0.01 to 1.19 ± 0.02 N and 0.65 ± 0.04, respectively. Hardness is a quantitative measurement of gel strength and structural stability, whereas cohesiveness represents the ability of the gel network to maintain structural integrity. These properties are closely related to the extent of protein cross-linking. The observed increase in hardness and cohesiveness with increasing PHP concentration (0%–2.0%) suggested that a higher level of PHP was more conducive to enhancing the protein aggregation and cross-linking, thereby facilitating the formation of more compact and well-constructed protein network structures with a firm texture. The springiness of the PPI-2.0% PHP composite gel (4.22 ± 0.04 mm) was approximately 1.5 times that of the PPI gel (2.85 ± 0.07 mm). The significant increase in springiness suggested that the presence of PHP enhanced the capacity of PPI-PHP composite gels to withstand deformation and reduced the susceptibility to fragmentation under external forces. This result may be attributed to the superior water-binding capacity of PHP. Specifically, higher amounts of PHP resulted in increased water retention and elevated hydration levels within the gel network, thereby contributing to greater resistance to deformation. It was noteworthy that the chewiness was incredibly enhanced by 2% PHP, increasing from 0.72 ± 0.04 mJ to 3.28 ± 0.15 mJ, which was a 3.5-fold increase compared to that of PPI gel. The pronounced increase in chewiness by PHP might be explained by the hydrophilic properties and long-chain molecular structures of PHP that enabled it to interact with protein molecules in the gel matrix through physical entanglement, hydrogen bonding, and electrostatic interactions. These interactions contributed to the formation of a more extensive and interconnected network within the composite gel structure, thereby increasing the energy required to fully masticate the gel. However, when the PHP concentration reached 3.0%, the hardness, springiness, chewiness, and cohesiveness all decreased, which was consistent with the previous studies about the impacts of PHP on the textural properties of the myofibrillar protein [12,16], as well as other excess polysaccharides (gum arabic, pectin, and hyaluronic acid) on protein gels [5,28,29]. These reduction trends implied that the high content incorporation of PHP may lead to phase separation between the protein and PHP, thereby disrupting the continuity of protein network structures. Overall, the TPA results indicated that the incorporation of PHP systematically improved the textural attributes of the PPI-PHP composite gels in a dose-dependent manner, with an optimal PHP addition suggested at 2.0%.

### 3.4. Macrostructure and Microstructure of PPI-PHP Composite Gels

**Figure 2:**
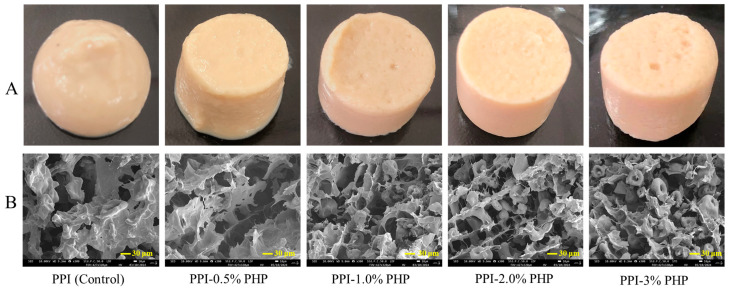
Optical (**A**) and scanning electron microscopy (**B**) images of heat-induced PPI-PHP composite gels.

The visual appearance of the gel provides insight into its macroscopic molding state, whereas the inner microstructure reflects the pore size and the three-dimensional network structure. These properties are closely related to the WHC and texture characteristics of the gels [30]. As shown in Figure 2, the heat-induced PPI gel without PHP exhibited a soft and mush-like appearance with a loose network framework that contained irregular and large pores within its microstructure. In contrast, the network architecture of the composite gel with 0.5% PHP exhibited slight interconnectivity, and the appearance showed the potential for self-standing. As the concentration of PHP gradually increased from 0.5% to 2.0%, the microstructure of the composite gels became increasingly homogeneous, with finer connections and smaller cavities. Concurrently, the appearance became smoother, firmer, and denser. A finely structured and highly interconnected gel network with uniform honeycomb structures could be observed in PPI-2.0% PHP composite gels. This result indicates that the presence of PHP facilitated the molecular rearrangement and crosslinking of unfolded proteins, thereby generating an ordered and homogeneous structure. This microstructure with increased porosity and homogeneity had the potential to hold more water, which was in accordance with the results of the WHC. When the amount of PHP was increased from 2.0% to 3.0%, the microstructure became more random and less coherent, and the macrostructures displayed more and larger pores as compared to the gel with 2.0% PHP. Likewise, Zhou et al. [16] reported that the addition of psyllium husk at 3.0% (*w*/*w*) led to the disruption of the myofibrillar network and the deterioration of textural properties due to the dominate role of the polysaccharide network in the mixed system. According to Yang, et al. [31], at high concentrations of polymers, intermolecular interactions between two polymers were not as favorable as interactions between similar polymer chain segments, which resulted in a tendency for the two polymers to exclude with each other and caused thermal incompatibility and phase separation. Therefore, the coarse network structures with 3.0% PHP may result from the steric hindrance of the PHP and the phase separation between the macromolecules, which consequently blocked the continuity of the three-dimensional gel networks. Overall, these results suggested that the incorporation of PHP, especially at the addition level of 2.0%, contributed to gel formation and enhancement by increasing the continuity of the protein gel network and promoting the compactness of the gel structures.

### 3.5. Rheological Properties

**Figure 3:**
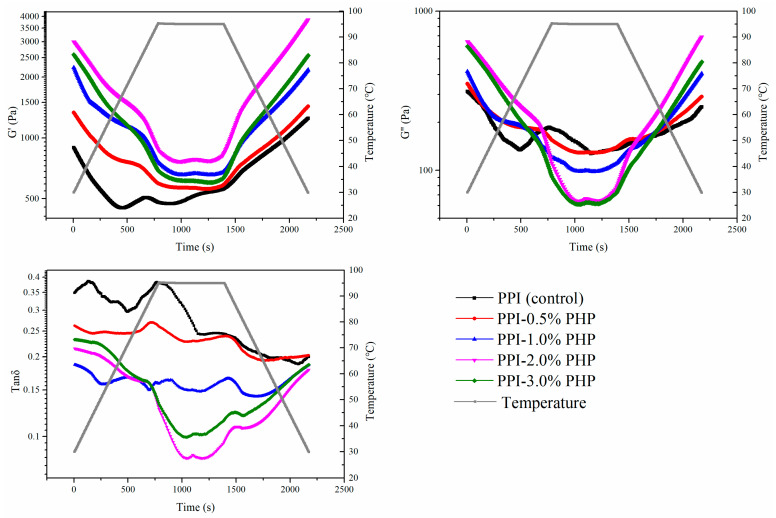
Changes of the storage modulus (G′), loss modulus (G″), and loss angle (tan δ) in temperature sweep for the dispersions of PPI-PHP mixtures.

Dynamic temperature sweep tests were conducted to reflect the kinetics of water migration, molecular interactions, protein aggregations, and gel network construction [17]. The G′ value serves to characterize alterations in gel-forming capacity and structural integrity and stiffness, as well as the interaction intensity between atoms, molecules, or ions at a microscopic level. Conversely, the G″ value quantifies the energy dissipated during deformation and relaxation, thereby signifying the presence of internal friction and molecular mobility within the gel network [32]. As shown in Figure 3, the G′ and G″ values of PPI-PHP mixtures decreased gradually from 30 °C to 95 °C, remained relatively constant during isothermal holding at 95 °C, and then increased sharply during the subsequent cooling period. The initial decrease trends can be explained by the dissociation and disruption of molecular interactions (ionic and hydrogen bonds) among proteins as the temperature rose, resulting in a decrease in elastic behavior. The exponential increase in G′ during cooling was the result of a reduction in entropy, which enhanced the attractive forces and contributed to the cross-linking and aggregation of protein molecules, thus generating an irreversible elastic three-dimensional network structure. Compared with the control group, the addition of PHP remarkably increased the modules during both the heating and cooling phases. As the PHP content varied from 0% to 3.0%, the G′ and G″ curves reached maximum values at the 2.0% level, and then decreased at the 3.0% level. This was consistent with the texture results. It could be explained that PHP within an appropriate range functioned as a filler to increase the number of cross-linked sites in the gel network, thereby improving the resistance to external forces. Moreover, the chain entanglement of the denatured proteins and PHP played roles as bridges of the polymer network. The enhanced hydrophobic interactions, hydrogen bonding, and electrostatic interactions (as shown in Section 3.8) between the unfolded proteins and PHP further enhanced the viscoelasticity of the composite gels. However, the high content of PHP (3%) absorbed water in a competitive manner, which partially restrained the swelling of proteins and hindered their stretching and aggregation. The self-aggregation of PHP would result in increased steric hindrance and form physical barriers within the protein network. These interferences synergistically hindered effective molecular interactions, leading to a reduction in the overall crosslinking density and mechanical strength of the composite gels.

The values of tan δ were less than 1 across the entire temperature range, indicating that the PPI-PHP mixtures exhibited highly solid-like behavior. The lower tan δ values of PPI-PHP mixtures than that of PPI implies the formation of a more energy-stable structure with longer-lasting intermolecular bonds in the composite gels [25]. It is noteworthy that there was an obvious peak at 95 °C in the tan δ curve of PPI, which might be related to the association and aggregation of the pea protein molecules. However, as the additional content of PHP increased from 0.5% to 3.0%, this peak gradually weakened and disappeared. This result indicates that the presence of PHP effectively hindered the rapid and random aggregation of protein during thermal processing by strong electrostatic repulsion, and provided sufficient time for protein unfolding, orientation, rearrangement, and crosslinking, thus developing a well-organized and firmer protein network.

### 3.6. Surface Hydrophobicity

**Figure 4:**
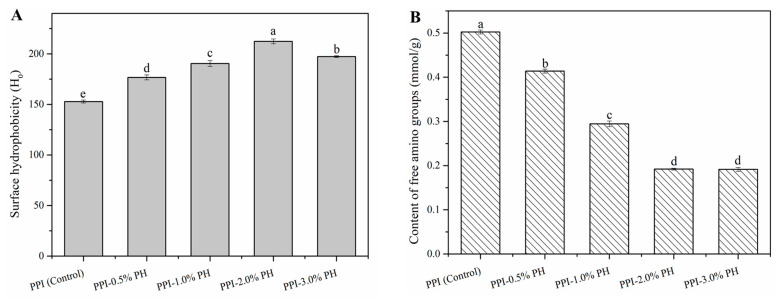
The surface hydrophobicity (**A**) and the content of free amino groups (**B**) of heat-induced PPI-PHP composite gels. Different letters at the top of the columns represent significant differences (*p* < 0.05) in varying amounts of PHP.

To gain further insight into the effects of PHP on the surrounding microenvironment and the conformational changes of PPI, the surface hydrophobicity was determined. As shown in Figure 4A, the surface hydrophobicity of the composite gels significantly increased (*p* < 0.05) as the PHP concentration increased from 0% to 2.0%. The surface hydrophobicity of the PPI- 2.0% PHP composite gels was up to 212.4 ± 2.4, representing a 38.90% increase compared with the control group (152.9 ± 1.5). This result may be because PHP containing large amounts of hydroxyl groups could compete with proteins for water molecules. This competition led to an increase in the hydrophobicity of the surrounding environment, thereby promoting the exposure of non-polar amino acid residues of proteins. The physical entanglements between the heat-induced unfolding protein molecular chains and PHP further contributed to the maintenance of high exposure levels of hydrophobic groups. Moreover, given that the isoelectric point of PPI was around a pH of 4.5, most of the amino acids in PPI were negatively charged in the neutral environment [33]. PHP, an anionic polysaccharide with a negative charge from ionized carboxyl groups, would generate strong electrostatic repulsive forces between PHP and PPI, thereby promoting the exposure of hydrophobic residues [34]. This finding was consistent with the previous reports about the impacts of other anionic polysaccharides (carrageenan and gum arabic) on the surface hydrophobicity of soybean protein gel and myofibrillar protein gel [35,36]. However, when the PHP concentration was raised to 3.0%, the surface hydrophobicity decreased to 197.4 ± 0.8. This result might result from the hydrogen bonding between PHP and the carboxyl and amino groups on proteins, which generated a hydrophilic boundary surrounding the protein molecules, consequently shielding the available binding sites for fluorescent probes. Furthermore, at high concentrations, the excluded volume and spatial steric hindrance effect amongst macromolecular biopolymers may lead to the partial refolding of hydrophobic side chains within the interior of proteins.

### 3.7. Content of Free Amino Groups

The variation in the content of free amino groups serves as a sensitive indicator of structural modifications and chemical interactions within the gel matrix [19]. As can be seen from Figure 4B, the incorporation of PHP dramatically reduced the content of free amino groups in PPI-PHP composite gels. As the PHP content increased from 0% to 2.0%, the content of free amino groups decreased significantly from 0.502 ± 0.004 mmol/g to 0.192 ± 0.002 mmol/g, representing a loss of approximately 62% loss. This result may be attributed to the concentrated effect of PHP through absorbing the moisture around the protein, as well as the improvement effect of PHP on the exposure degree of protein molecular chains (as indicated by the results of surface hydrophobicity). These effects facilitated the self-aggregation and crosslinking of protein molecules, thereby reducing the number of available amino groups. Moreover, as the content of PHP increased, a greater number of available hydroxyl groups were induced to react with free amino groups on proteins through hydrogen bonding, which was expected to further reduce the value. However, when the PHP content exceeded 2.0%, there was no additional reduction in free amino groups. This phenomenon suggests that PHP at the 3.0% level in the composite gels may have exceeded the saturation point where all available amino groups had formed cross-links with adjacent proteins and PHP, thereby preventing further reduction in the content of free amino groups.

### 3.8. Molecular Interaction Forces

**Figure 5:**
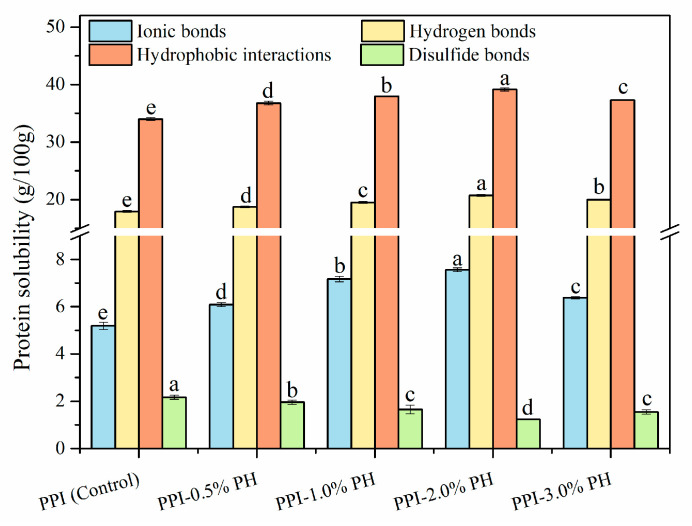
Molecular interaction forces of heat-induced PPI-PHP composite gels. Different letters at the top of the columns represent significant differences (*p* < 0.05) in varying amounts of PHP.

The three-dimensional network structure, textural properties, and stability of protein gels are essentially dependent on the construction and maintenance of various intermolecular forces. As shown in Figure 5, the intensity of the chemical forces in heat-induced PPI and PPI-PHP gels were in the order of hydrophobic interactions > hydrogen bonds > ionic bonds > disulfide bonds. It indicated that hydrophobic interactions and hydrogen bonds were the major molecular driving forces for the formation and maintenance of the PPI gel structure, which was consistent with the previous reports by Kornet, et al. [37] and Faber, et al. [38]. According to Zhang, et al. [39], the minor contribution of disulfide bonds to the stabilization of a pea protein gel was due to the lack of free SH groups and the limited content of cysteine. With the increase in PHP concentration from 0% to 2.0%, the hydrophobic interactions, hydrogen bonding, and electrostatic interactions were all significantly enhanced, which was responsible for the improved texture. Among them, the contribution of hydrophobic interactions to the protein solubility exhibited the most pronounced increase, rising from 34.00 ± 0.24 g/100 g to 39.16 ± 0.27 g/100 g. Similarly, previous studies [40,41] reported that the addition of κ-carrageenan, sodium alginate, and xanthan gum significantly promoted hydrophobic interactions between soy protein molecules. This was due to the hydrophilic nature and abundance of carboxyl and hydroxyl groups on polysaccharides, which enabled them to induce the exposure of hydrophobic groups and absorb moisture, thus promoting hydrophobic interactions and the further aggregation of proteins [40]. Moreover, the increase in PHP concentration introduced a large number of hydroxyl groups which could form hydrogen bonding with the carboxyl and amino groups of PPI. The hydrogen bonding between PHP and PPI altered the spatial barriers and polar environment around proteins, which induced the exposure of hydrophobic groups and further enhanced the hydrophobic interactions [41]. The anionic PHP with a negative charge from ionized carboxyl groups could interact with positively charged groups (NH_3_^+^) on proteins through electrostatic interactions. This enabled PHP to act as electrostatic bridges between cationic groups on different protein molecules. However, there was a slight decrease in the disulfide bond as the PHP concentration increased from 0% to 2.0%. This phenomenon might be due to the heat-induced protein unfolding and the breakage of the disulfide bond. The entanglement and molecular interactions between pea protein and PHP presumably maintained the stretching structure of proteins and the reduced spatial accessibility of the cysteine residues, thereby reducing the possibility of the formation of S-S bonds. The high content of PHP (3.0%) weakened all interaction forces within the composite gels, which was responsible for the decreased textural properties. This may be because the self-aggregation of PHP in the composite gels hindered intermolecular contacts and reduced the probability of protein aggregation and crosslinking. Therefore, the gel-strengthening effect of PHP, especially at a level of 2.0%, was mainly attributed to the enhanced hydrophobic interactions, hydrogen bonding, and electrostatic interactions within the gel networks.

### 3.9. SDS-PAGE Profiles

**Figure 6:**
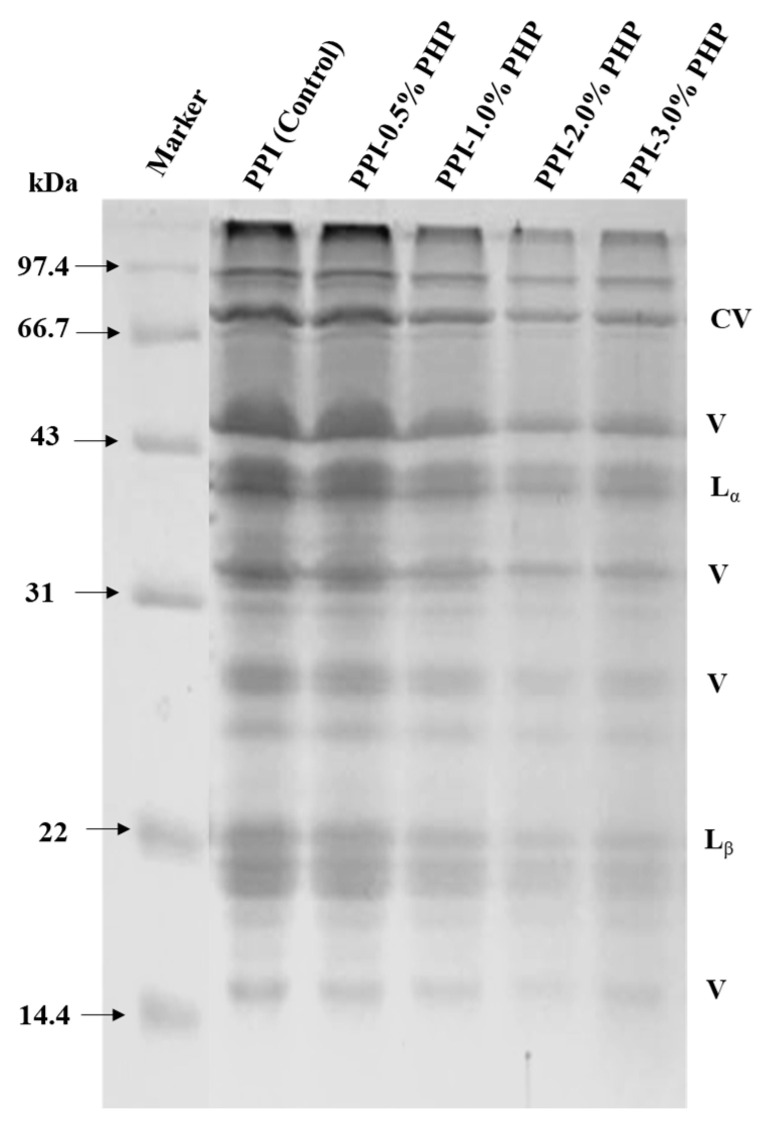
Sodium dodecyl sulfate polyacrylamide gel electrophoresis (SDS-PAGE) pattern of heat-induced PPI-PHP composite gels. CV stands for con-vicilin, V for vicilin, L_α_ for legumin acidic subunit, and L_β_ for legumin basic subunit.

To obtain insight into the effect of PHP on the aggregation behavior and subunit profile of pea protein, electrophoresis was performed. The components of pea protein were commonly categorized into three major groups, namely, legumin (11S, ~40 kDa and 20 kDa), vicilin (7S, ~55 kDa, 30 kDa, and <19 kDa), and convicilin (7S, ~70 kDa) [42]. As shown in Figure 6, after heat induction to form a gel with the addition of PHP, legumin, and vicilin remained the main components of pea protein without any new individual protein bands generated in the gel pattern. This suggests that the addition of PHP did not cause changes in the peptide chain structure of pea protein. This observation might be in response to the fact that pea protein mainly interacts with PHP through non-covalent bonding and physical entanglements [43]. With the PHP content increasing from 0.5% to 2.0%, the imprints of the bands became noticeably narrowed and lightened. This observation was assumed as a consequence of the increased surface hydrophobicity by PHP that reduced the solubility of pea protein. Moreover, the PHP encasing the outer layer of protein molecules reduced the binding sites of protein molecules to Coomassie brilliant blue. When PHP was added at the level of 3.0%, the band strength slightly increased, indicating that the high content of PHP weakened the polymerization and cross-linking of protein and partially increased the soluble fractions. Therefore, the incorporation of PHP changed the unfolding, rearrangement, aggregation, and crosslinking behaviors of legumins and vicilins, which further regulated the construction of gel network structures.

### 3.10. Secondary Structural Changes

**Figure 7:**
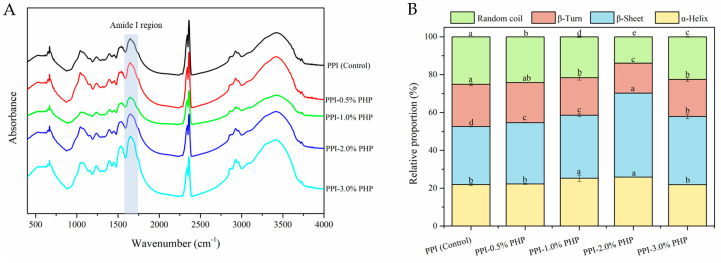
Fourier-transform infrared spectra of PPI−PHP composite gels in the range of 400−4000 cm^−1^ (**A**) and the relative proportion of secondary structures (**B**). Different letters in the columns represent significant differences (*p* < 0.05) in varying amounts of PHP.

FTIR is commonly used to provide information about alterations in protein functional groups and the relative content of the secondary structures. As shown in Figure 7A, after PHP was added, the infrared spectra of all the samples showed similar skeletons without the formation of new peaks, suggesting that PPI mainly combined with PHP through non-covalent interactions without generating new covalent bonds. This finding was in good agreement with the results of SDS-PAGE gel electrophoresis and molecular interaction forces.

The relative content of protein secondary structures (Figure 7B) can be calculated by deconvoluting the amide I band (1600–1700 cm^−1^) and fitting the second derivative spectrum using Gaussian functions. The curve-fitted spectra of the amide I band were assigned as α-helix (1646–1664 cm^−1^), β-sheet (1615–1637 cm^−1^ and 1682–1700 cm^−1^), β-turn (1664–1681 cm^−1^), and random coil (1637–1645 cm^−1^) [6]. As the PHP content increased from 0% to 2.0%, the relative proportion of α-helix increased slightly from 21.929 ± 0.578% to 25.886 ± 0.004%, whereas β-sheet structures increased significantly from 30.700 ± 0.202% to 44.384 ± 0.022%, accompanied by the decreases in β-turn and random coil from 22.263 ± 0.674% and 25.108 ± 0.106% to 15.850 ± 0.016% and 13.879 ± 0.002%, respectively. Previous studies have reported that α-helix structure is mainly associated with the stability of the hydrogen bonds between carbonyl oxygen (-CO) and amino hydrogen (-NH) in the polypeptide chain [43,44]. The intermolecular hydrogen-bonded β-sheet acted as junction zones to stabilize the gel network, thus the content of the β-sheet structure was positively correlated with the degree of protein aggregation, as well as the thermal stability, textural properties, and the WHC of the gel [43,44]. The β-turn and random coil structures were adversely related to the organization of the ordered gel network structure due to their relatively irregular and loose shape [45]. Therefore, the transformation of β-turn and random coil to α-helix and β-sheet structures may be due to the fact that PHP with a large number of hydroxyl groups facilitated the formation of intermolecular and intramolecular hydrogen bonds, which was advantageous for maintaining protein conformational stability and the organization of a more ordered and cross-linked gel network structures. According to Sasidharan and Ramakrishnan [46], β-sheet structures with an extended backbone permitted hydrogen bonding between the backbone amides and carbonyls, as well as the formation of long-range interactions. The intermolecular repulsion between anionic PHP and negatively charged PPI in a neutral environment facilitated the unfolding of peptide chains during heating and the establishment of hydrogen bonds between neighboring groups after cooling, thereby leading to the generation of more β-sheet structures [47]. However, the addition of PHP at a 3.0% level resulted in increases in the relative content of β-turn and random coil with concomitant decreases in the content of α-helix and β-sheet structures. The loss of highly ordered secondary structures was attributed to the weakened hydrogen bonding and hydrophobic interactions among protein groups. It might be because the high concentration of PHP inhibited the ordered rearrangement of unfolding protein chains and blocked the cross-linking of protein molecules, thus leading to a looser and more disordered protein structure. Therefore, it can be concluded that the substantial increase in the relative content of β-sheet structures predominantly contributed to the enhancement of gel strength.

### 3.11. Mechanistic Explanation

**Figure 8:**
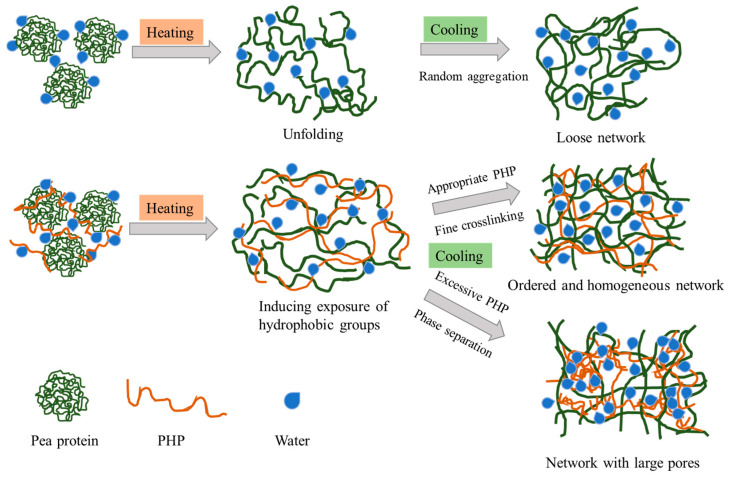
A schematic diagram of the influence mechanism of PHP on the heat-induced PPI gelation.

Based on the above results, the underlying mechanism and schematic model of the enhancement effect of PHP on the pea protein gelation properties are shown in Figure 8. After heating treatment, the denatured pea protein exposed the active residues that were initially buried inside and then aggregated randomly to build up a disordered and coarse gel network with weak strength, which was proven by the results of SEM, texture analysis, and rheological properties. Moderate levels of PHP (0.5–2.0%) played roles as active filler and an effective water binder which created concentrated conditions for pea protein and decreased the intermolecular distance between proteins, providing a greater chance for protein aggregation and crosslinking [48]. Moreover, the entanglement of unfolding pea proteins and PHP facilitated the maintenance of high exposure levels of hydrophobic groups and enhanced hydrophobic interactions. Additionally, the hydrogen bonding between the hydroxyl groups of PHP and the carboxyl and amino groups of PPI further contributed to the establishment of ordered protein structures and improvement of gel properties. The strong electrostatic repulsion between anionic PHP and negatively charged PPI in a neutral environment effectively prevented the rapid and random aggregation of proteins and provided sufficient time for protein swelling, complete unfolding, rearrangement, aggregation, and crosslinking [49]. This consequently resulted in the construction of a well-organized and fine gel network with a higher WHC and gel strength. However, a high concentration of PHP (3.0%) tended to self-aggregate, which disrupted the integrity and connectivity of the protein gel network. Moreover, thermodynamic incompatibility and phase separation between protein and polysaccharides at high concentrations led to steric hindrance for protein cross-linking, thus resulting in an inhomogeneous structure and weakened textural properties [50]. Overall, the incorporation of PHP altered the water distribution, rheological properties, intermolecular interaction forces, spatial conformations, unfolding process, and aggregation kinetics of protein molecules, which jointly contributed to the regulation and improvement of gel properties.

## 4. Conclusions

In the present study, the effects of PHP with different addition levels on the physicochemical, structural, and gelation properties of pea protein were investigated. The results showed that the incorporation of PHP at a level of 0.5–2.0% significantly improved the textural properties, WHC, whiteness, and G′ and G″ values of heat-induced pea protein gel. The incorporation of PHP significantly increased the surface hydrophobicity and enhanced hydrophobic interactions, hydrogen bonding, and electrostatic interactions in composite gels, and prevented the rapid and random aggregation of proteins by electrostatic repulsions. Moreover, PHP facilitated the transformation of β-turn and random coil to α-helix and β-sheet structures, thereby contributing to the formation of an ordered and highly interconnected gel network with uniform honeycomb structures, and a smooth appearance. However, local gelation and self-aggregation of excessive PHP (3.0%) partially weakened effective molecular interactions and interrupted the integrity of protein network structures, leading to the formation of rough and less coherent microstructures and slight reductions in the WHC and gel strength. Overall, PHP, especially at a level of 2.0%, could be used as an effective natural gel modifier in improving the gelation properties of pea proteins. This study provided valuable insight and in-depth understanding for developing and tailoring innovative plant protein-based foods with strong gel properties, good water binding capacity, and enriched dietary fiber content. To fully validate the practical applications of PPI-PHP composite gels in various foods, such as plant-based meat analogs, fat substitutes, cheese, yogurt, sausages, jams, desserts, jellies, confectioneries, etc., future work will conduct sensory evaluations, nutritional value assessment, the determination of digestive properties, and an investigation on the stability at different temperatures, pH levels, or ionic strengths.

## Figures and Tables

**Table 1:** The effects of psyllium husk powder (PHP) content on the color attributes and water holding capacity (WHC) of heat-induced pea protein isolate (PPI)gel.

Sample	L*	a*	b*	W	WHC (%)
PPI	50.20 ± 0.26 ^d^	2.17 ± 0.07 ^b^	14.04 ± 1.38 ^a^	48.20 ± 0.19 ^c^	78.4 ± 0.7 ^e^
PPI-0.5%-PHP	73.34 ± 0.90 ^b^	2.28 ± 0.06 ^ab^	18.86 ± 0.36 ^b^	67.26 ± 0.75 ^a^	81.6 ± 0.3 ^d^
PPI-1.0%-PHP	74.22 ± 0.97 ^b^	2.33 ± 0.08 ^a^	19.28 ± 0.45 ^b^	67.72 ± 0.95 ^a^	86.3 ± 0.8 ^c^
PPI-2.0%-PHP	76.21 ± 0.15 ^a^	2.37 ± 0.03 ^a^	20.07 ± 0.14 ^b^	68.78 ± 0.12 ^a^	92.6 ± 1.0 ^a^
PPI-3.0%-PHP	71.13 ± 1.58 ^c^	2.30 ± 0.06 ^a^	19.97 ± 0.18 ^b^	64.81 ± 1.38 ^b^	89.1 ± 0.6 ^b^

Note: Different letters in the same column mean significant differences (*p* < 0.05) in varying amounts of PHP.

**Table 2:** The effects of PHP content on the textural properties of heat-induced PPI gel.

Sample	Hardness (N)	Cohesiveness	Springiness (mm)	Chewiness (mJ)
PPI	0.65 ± 0.02 ^e^	0.39 ± 0.01 ^d^	2.85 ± 0.07 ^c^	0.72 ± 0.04 ^e^
PPI-0.5%-PHP	0.70 ± 0.02 ^d^	0.46 ± 0.03 ^c^	3.61 ± 0.03 ^b^	1.17 ± 0.07 ^d^
PPI-1.0%-PHP	1.06 ± 0.04 ^c^	0.56 ± 0.02 ^b^	3.77 ± 0.05 ^b^	2.22 ± 0.19 ^c^
PPI-2.0%-PHP	1.19 ± 0.02 ^a^	0.65 ± 0.04 ^a^	4.22 ± 0.04 ^a^	3.28 ± 0.15 ^a^
PPI-3.0%-PHP	1.14 ± 0.02 ^b^	0.62 ± 0.02 ^a^	3.74 ± 0.15 ^b^	2.65 ± 0.13 ^b^

Note: Different letters in the same column mean significant differences (*p* < 0.05) in varying amounts of PHP.

## Data Availability

The original contributions presented in the study are included in the article, further inquiries can be directed to the corresponding author.

## References

[1] Chen Q., Zhang J., Zhang Y., Meng S., Wang Q. (2021). Rheological properties of pea protein isolate-amylose/amylopectin mixtures and the application in the high-moisture extruded meat substitutes. Food Hydrocoll..

[2] Zhang J., Xu H., Liu H., Wang W., Zheng M., Liu Y., Zhou Y., Li Y., Sui X., Xiao Y. (2024). Insight into the improvement mechanism of gel properties of pea protein isolate based on the synergistic effect of cellulose nanocrystals and calcium ions. Food Chem..

[3] Ge J., Sun C.-X., Corke H., Gul K., Gan R.-Y., Fang Y. (2020). The health benefits, functional properties, modifications, and applications of pea (*Pisum sativum* L.) protein: Current status, challenges, and perspectives. Compr. Rev. Food Sci. Food Saf..

[4] Boukid F., Rosell C.M., Castellari M. (2021). Pea protein ingredients: A mainstream ingredient to (re)formulate innovative foods and beverages. Trends Food Sci. Technol..

[5] Wang K., Sun H., Cui Z., Wang J., Hou J., Lu F., Liu Y. (2024). Synergistic effects of microbial transglutaminase and apple pectin on the gelation properties of pea protein isolate and its application to probiotic encapsulation. Food Chem..

[6] Li Y., Qi X., Rong L., Li J., Shen M., Xie J. (2024). Effect of gellan gum on the rheology, gelling, and structural properties of thermally induced pea protein isolate gel. Food Hydrocoll..

[7] Cortez-Trejo M.C., Gaytán-Martínez M., Reyes-Vega M.L., Mendoza S. (2021). Protein-gum-based gels: Effect of gum addition on microstructure, rheological properties, and water retention capacity. Trends Food Sci. Technol..

[8] Ryu J., Xiang X., Hu X., Rosenfeld S.E., Qin D., Zhou H., McClements D.J. (2023). Assembly of plant-based meat analogs using soft matter physics: A coacervation-shearing-gelation approach. Food Hydrocoll..

[9] Xu Q., Qi B., Han L., Wang D., Zhang S., Jiang L., Xie F., Li Y. (2021). Study on the gel properties, interactions, and pH stability of pea protein isolate emulsion gels as influenced by inulin. LWT-Food Sci. Technol..

[10] He Z., Liu C., Zhao J., Li W., Wang Y. (2021). Physicochemical properties of a ginkgo seed protein-pectin composite gel. Food Hydrocoll..

[11] Gao J., Zhu S., Lv S., Xu J., Zheng M., Liu Y., Zhou Y., Song C., Sui X., Xiao Y. (2024). Dose-effect relationship and molecular mechanism of cellulose nanocrystals improving the gel properties of pea protein isolate. Food Hydrocoll..

[12] Bi J., Sun Y., Pan D., Zhou C., Du L. (2024). Effect of ultrasound combined with psyllium husk powder on the structure and gel properties of goose myofibrillar protein. Process Biochem..

[13] Franco E.A.N., Sanches-Silva A., Ribeiro-Santos R., de Melo N.R. (2020). Psyllium (Plantago ovata Forsk): From evidence of health benefits to its food application. Trends Food Sci. Technol..

[14] Fu Q.-q., Liu R., Zhou L., Zhang J.-w., Zhang W.-g., Wang R.-r. (2022). Effects of psyllium husk powder on the emulsifying stability, rheological properties, microstructure, and oxidative stability of oil-in-water emulsions. Food Control.

[15] Zhu N., Zang M., Wang S., Zhang S., Zhao B., Liu M., Li S., Wu Q., Liu B., Zhao Y. (2022). Modulating the structure of lamb myofibrillar protein gel influenced by psyllium husk powder at different NaCl concentrations: Effect of intermolecular interactions. Food Chem..

[16] Zhou Y., Dai H., Ma L., Yu Y., Zhu H., Wang H., Zhang Y. (2021). Effect and mechanism of psyllium husk (Plantago ovata) on myofibrillar protein gelation. LWT-Food Sci. Technol..

[17] Feng Y., Liang X., Zhao Z., Kong B., Xia X., Cao C., Zhang H., Liu Q., Sun F. (2024). Mechanisms and effects of different crosslinking degrees on gelling properties and in vitro digestibility of heat-induced microbial transglutaminase-mediated myofibrillar protein gels with or without κ-carrageenan. Food Hydrocoll..

[18] Sun Y., Wang L., Wang H., Zhou B., Jiang L., Zhu X. (2025). Effect of pH-shifting and ultrasound on soy/potato protein structure and gelation. Food Hydrocoll..

[19] Liao X., Xu J., Lv S., Zhu S., Wang W., Zhou Y., Liu Y., Sui X., Xiao Y. (2024). Theanine improves the gelation of soy protein isolate by modifying protein conformation and enhancing molecular interaction. Food Hydrocoll..

[20] Zhao Y., Wei K., Chen J., Wei G., Li J., Zheng B., Song Y., Gao P., Zhou R. (2024). Enhancement of myofibrillar protein gelation by plant proteins for improved surimi gel characteristics: Mechanisms and performance. LWT-Food Sci. Technol..

[21] Wojciechowicz-Budzisz A., Pejcz E., Spychaj R., Harasym J. (2023). Mixed Psyllium Fiber Improves the Quality, Nutritional Value, Polyphenols and Antioxidant Activity of Rye Bread. Foods.

[22] Lv Y., Zhao H., Xu Y., Yi S., Li X., Li J. (2023). Properties and microstructures of golden thread fish myofibrillar proteins gel filled with diacylglycerol emulsion: Effects of emulsifier type and dose. Food Hydrocoll..

[23] Guidi S., Formica F.A., Denkel C. (2022). Mixing plant-based proteins: Gel properties of hemp, pea, lentil proteins and their binary mixtures. Food Res. Int..

[24] Khemakhem M., Attia H., Ayadi M.A. (2019). The effect of pH, sucrose, salt and hydrocolloid gums on the gelling properties and water holding capacity of egg white gel. Food Hydrocoll..

[25] Moreno H.M., Domínguez-Timón F., Díaz M.T., Pedrosa M.M., Borderías A.J., Tovar C.A. (2020). Evaluation of gels made with different commercial pea protein isolate: Rheological, structural and functional properties. Food Hydrocoll..

[26] Klost M., Brzeski C., Drusch S. (2020). Effect of protein aggregation on rheological properties of pea protein gels. Food Hydrocoll..

[27] Noguerol A.T., Larrea V., Pagán M.J. (2022). The effect of psyllium (*Plantago ovata* Forsk) fibres on the mechanical and physicochemical characteristics of plant-based sausages. Eur. Food Res. Technol..

[28] Ma C., Li S., Yin Y., Xu W., Xue T., Wang Y., Liu X., Liu F. (2022). Preparation, characterization, formation mechanism and stability of allicin-loaded emulsion gel. LWT-Food Sci. Technol..

[29] Wang N., Zhang K., Chen Y., Hu J., Jiang Y., Wang X., Ban Q. (2023). Tuning whey protein isolate/hyaluronic acid emulsion gel structure to enhance quercetin bioaccessibility and in vitro digestive characteristics. Food Chem..

[30] Ren C., Hong S., Qi L., Wang Z., Sun L., Xu X., Du M., Wu C. (2023). Heat-induced gelation of SAM myofibrillar proteins as affected by ionic strength, heating time and temperature: With emphasis on protein denaturation and conformational changes. Food Biosci..

[31] Yang X., Li A., Li D., Guo Y., Sun L. (2021). Applications of mixed polysaccharide-protein systems in fabricating multi-structures of binary food gels—A review. Trends Food Sci. Technol..

[32] Huang C., Blecker C., Wei X., Xie X., Li S., Chen L., Zhang D. (2024). Effects of different plant polysaccharides as fat substitutes on the gel properties, microstructure and digestion characteristics of myofibrillar protein. Food Hydrocoll..

[33] Guldiken B., Saffon M., Nickerson M.T., Ghosh S. (2023). Improving physical stability of pea protein-based emulsions near the isoelectric point via polysaccharide complexation. Food Hydrocoll..

[34] Belorio M., Gómez M. (2021). Psyllium: A useful functional ingredient in food systems. Crit. Rev. Food Sci..

[35] Zhou H., Hu X., Xiang X., McClements D.J. (2023). Modification of textural attributes of potato protein gels using salts, polysaccharides, and transglutaminase: Development of plant-based foods. Food Hydrocoll..

[36] Lin S., Liang X., Zhao Z., Kong B., Cao C., Sun F., Liu Q. (2024). Elucidating the mechanisms of ultrasound treatment combined with κ-carrageenan addition enhancing the gelling properties of heat-induced myofibrillar protein gel. Food Res. Int..

[37] Kornet R., Veenemans J., Venema P., van der Goot A.J., Meinders M., Sagis L., van der Linden E. (2021). Less is more: Limited fractionation yields stronger gels for pea proteins. Food Hydrocoll..

[38] Faber I., Pouvreau L., Jan van der Goot A., Keppler J. (2024). Modulating commercial pea protein gel properties through the addition of phenolic compounds. Food Hydrocoll..

[39] Zhang D., Chen D., Patel B., Campanella O.H. (2022). Pectin as a natural agent for reinforcement of pea protein gel. Carbohyd. Polym..

[40] Wang J., Jiang Q., Huang Z., Muhammad A.H., Gharsallaoui A., Cai M., Yang K., Sun P. (2024). Rheological and mechanical behavior of soy protein-polysaccharide composite paste for extrusion-based 3D food printing: Effects of type and concentration of polysaccharides. Food Hydrocoll..

[41] Wang Z., Long J., Zhang C., Hua Y., Li X. (2025). Effect of polysaccharide on structures and gel properties of microgel particle reconstructed soybean protein isolate/polysaccharide complex emulsion gels as solid fat mimetic. Carbohyd. Polym..

[42] Chen Q., Zhang J., Zhang Y., Liu H., Li T., Wang Q., Kaplan D.L. (2023). Microscopic insight into the interactions between pea protein and fatty acids during high-moisture extrusion processing. Food Chem..

[43] Li X., Huang Q., Zhang Y., Huang X., Wu Y., Geng F., Huang M., Luo P., Li X. (2023). Study on the Mechanism of Modified Cellulose Improve the Properties of Egg Yolk gel. Food Chem. X.

[44] Ma Y., Wang Y., Jiang S., Zeng M. (2022). Effect of gelatin on gelation properties of oyster (*Crassostrea gigas*) protein. LWT-Food Sci. Technol..

[45] Mi H., Li Y., Wang C., Yi S., Li X., Li J. (2021). The interaction of starch-gums and their effect on gel properties and protein conformation of silver carp surimi. Food Hydrocoll..

[46] Sasidharan S., Ramakrishnan V., Donev R. (2022). Chapter Five—Aromatic interactions directing peptide nano-assembly. Advances in Protein Chemistry and Structural Biology.

[47] Cheng T., Wang Z., Sun F., Liu H., Liu J., Guo Z., Zhou L. (2024). Gel properties of rice proteins-pectin composite and the delivery potential for curcumin: Based on different concentrations and the degree of esterification of pectin. Food Hydrocoll..

[48] Feng J., Bai X., Li Y., Kong B., Nuerjiang M., Wu K., Li Z., Xia X. (2023). Improvement on gel properties of myofibrillar protein from chicken patty with potato dietary fiber: Based on the change in myofibrillar protein structure and water state. Int. J. Biol. Macromol..

[49] Chassenieux C., Nicolai T. (2024). Mechanical properties and microstructure of (emul)gels formed by mixtures of proteins and polysaccharides. Curr. Opin. Colloid Interface Sci..

[50] Zheng J., Van der Meeren P., Sun W. (2024). New insights into protein–polysaccharide complex coacervation: Dynamics, molecular parameters, and applications. Aggregate.

